# Molar Incisor Hypomineralization: An Epidemiological Study with Prevalence and Etiological Factors in Indian Pediatric Population

**DOI:** 10.5005/jp-journals-10005-1357

**Published:** 2016-06-15

**Authors:** Apurva Mishra, Ramesh K Pandey

**Affiliations:** 1 Junior Resident, Department of Pediatric and Preventive Dentistry, King George Medical University, Lucknow, Uttar Pradesh, India; 2 Head, Department of Pediatric and Preventive Dentistry, King George Medical University, Lucknow, Uttar Pradesh, India

**Keywords:** Enamel opacities, Molar incisal hypomineraliza-tion, Posteruptive breakdown, Prenatal and postnatal infections.

## Abstract

**Aims:** To determine the prevalence of molar incisor hypomineralization (MIH) in Indian children and to analyze the possible etiological factors.

**Materials and methods:** First permanent molars and all permanent incisors were examined in 1,369 children aged 8 to 12 years. Examinations were performed by two calibrated observers. The subjects were evaluated using judgment criteria proposed by Weerheijm et al in 2003. The parents accompanying children were given a questionnaire regarding pre- and postnatal history of the children.

**Results:** A total of 191 children were diagnosed with MIH with a prevalence of 13.9%. Chi-square/Fisher exact test was used to compare the dichotomous variables. The relative risk with its 95% confidence interval was calculated to find the risk of clinical infections, such as chicken pox, jaundice, renal disorders, cardiac disorders, and affected molars with sex and type of delivery. Pre- and postnatal history of infection in a child was significantly correlated with the prevalence of MIH.

**Conclusion:** The prevalence of MIH was 13.9% in the age group of 8 to 12 years. Prenatal and postnatal infections play an important role in hypomineralization of molars and incisors.

**How to cite this article:** Mishra A, Pandey RK. Molar Incisor Hypomineralization: An Epidemiological Study with Prevalence and Etiological Factors in Indian Pediatric Population. Int J Clin Pediatr Dent 2016;9(2):167-171.

## INTRODUCTION

Molar incisor hypomineralization (MIH) is a frequently noticed developmental defect found mainly in molars and incisors. Molar incisor hypomineralization is defined as the developmentally derived dental defect that involves hypomineralization of one to four permanent molars, frequently associated with similarly affected permanent incisors.^1^ The European Academy of Pediatric Dentistry Congress 2000 reported MIH with names, such as condition hypomineralized first permanent molar (FPM),^2^ idiopathic hypomineralization in FPM, cheese molar,^3^ and nonfluoride hypomineralization in FPM.^4^ Molar incisor hypomineralization clinical appearance may vary from white to yellow opacities to soft and porous enamel. The porous enamel subjected to masticatory stress leads to posteruptive breakdown of enamel, making tooth susceptible to thermal and cold stimuli. The various etiological factors for MIH depend on the prenatal, perinatal, and postnatal conditions of a child. The ameloblast during its secretory phase is sensitive to hypoxic conditions and temperature variation. Any disturbance during enamel formation leads to severe hypomineralization at the cusps of an affected tooth, with a well-defined border between the hypomineralized and normal enamel at the cervical third.^5^

Molar incisor hypomineralization is an important clinical problem of concern to a pediatric dentist due to its challenging management. The affected tooth are prone to dental caries with the lapse of time; more complex restorations in cooperative child and repeated breakdown of tooth and restoration may further aggravate the problem.

The purpose of the present study is to know the prevalence of MIH in children, to determine the possible etiological factors, and to compare the obtained data with its prevalence in other countries.

## MATERIALS AND METHODS

### Study Population

In the present study, a total of 1,369 children were examined in the age group of 8 to 12 years. The level of fluoride in the drinking water was optimal (0.7-1 ppm). The children with generalized hypoplastic/hypomineral-ized defects, such as amelogenesis imperfecta and those suffering from any chronic illness were excluded from the present study.

### Selection Criteria

All the children who participated in the present study were instructed to brush prior to the clinical examination. The status of permanent incisors and molars was evaluated and recorded, according to the judgment criteria.^6^ All the children diagnosed with MIH were reexamined by a second investigator to rule out any discrepancy regarding perception of judgment criteria. The entire indexed tooth was kept wet while examination to rule out opacities due to excessive drying, and the size of lesion was not taken into consideration. A set of questionnaires was given to the parents/guardian accompanying the 191 diagnosed children. The questionnaire contained a detailed history of mothers’ pregnancy, time of birth of child, and postnatal history of the child.

Molar incisor hypomineralization lesions were divided into three categories based on the severity of lesion, viz., opacities, posteruptive breakdown, and atypical restoration.

### Statistical Analysis

All the analyses were carried out using Statistical Package for the Social Sciences (SPSS) 16.0 version (Chicago, Inc., USA). Chi-square/Fisher exact test was used to compare the dichotomous variables. The relative risk (RR) with its 95% confidence interval (CI) was calculated to find the risk of MIH with gender distribution, aliments, such as jaundice, chest and ear infections, and type of delivery. The p-value < 0.05 was considered as significant.

**Table 1:** **Table 1:** Distribution of study population by gender, place of birth, and type of delivery

		*n = 191*		*Percentage*	
Age in years, mean ± SD		10.25 ± 1.25			
*Gender*					
Male		92		48.2	
Female		99		51.8	
*Place of birth*					
Rural		92		48.2	
Urban		99		51.8	
*Type of delivery*					
Normal		112		58.6	
Cesarean		79		41.4	

SD: Standard deviation

**Table 2:** **Table 2:** Distribution of clinical symptoms with gender

		*Total (n = 191)*				*Male (n = 92)*				*Female (n = 99)*					
		*No.*		*%*		*No.*		*%%*		*No.*		%		*RR (95% CI), p-value*	
Chicken pox		15		7.9		7		7.6		8		8.1		0.96 (0.55-1.69), 0.90	
Jaundice		12		6.3		7		7.6		5		5.1		1.22 (0.74-2.03), 0.46	
Cardiac problem		8		4.2		3		3.3		5		5.1		0.77 (0.31-1.91), 0.53	
Kidney problem		5		2.6		1		1.1		4		4.0		0.40 (0.07-2.37), 0.20	
Ear infection		9		4.7		7		7.6		2		2.0		1.66 (1.13-2.44), 0.04*	
Vitamin A deficiency		16		8.4		11		12.0		5		5.1		1.48 (1.02-2.14), 0.03*	
Chest infection		7		3.7		3		3.3		4		4.0		0.88 (0.37-2.11), 0.77	
High fever		19		9.9		10		10.9		9		9.1		1.10 (0.70-1.73), 0.68	
Intake of antibiotics		22		11.5		11		12.0		11		11.1		1.04 (0.66-1.63), 0.85	
Long-term breastfeeding		12		6.3		3		3.3		9		9.1		0.50 (0.18-1.35), 0.09	

RR: Relative risk; CI: Confidence interval; *Significant

**Graph 1:**
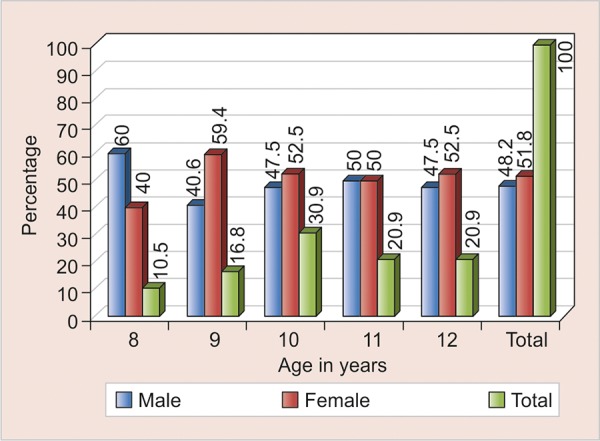
Age and gender distribution of children

## RESULTS

In total, 1,369 children aged 8 to 12 years were examined. Data of 191 children diagnosed with MIH were statistically analyzed. The interexaminer agreement was good (kappa = 0.872). A total of 48.2% male and 51.8% female children were diagnosed with MIH with mean age 10.25 ± 1.25 (mean ± standard deviation) (Table 1).

Distribution of the subjects by age and gender is presented in Graph 1. A total of 125 reported with a history of pre- and postnatal infection/antibiotic treatment. Based on the questionnaire, a correlation was established between probable etiology and occurrence of MIH. Vitamin A deficiency and ear infection in early childhood showed a marked and statistically significant correlation with MIH (p-value 0.03 and 0.04 respectively) (Table 2).

Prevalence of hypomineralized lesions was assessed; the majority of children were unaffected, whereas 191 (13.95%) had either molar/incisor or both affected with MIH. The children diagnosed were divided into three categories on the basis of tooth affected, viz., molars, molars with incisors, and incisors with opacities. The RR with its 95% CI was calculated to find the risk in molars or incisors or both and its distribution with gender (Tables 3 and 4) and type of delivery (Tables 5 and 6).

## DISCUSSION

Molar incisor hypomineralization is a common finding across the globe with a range of prevalence of 2 to 25%.^7,8^ This difference may be due to variation in ethnicity, sample size, and age of population in the preceding studies. The present study showed a prevalence of 13.9% in a group of Indian children, which is more than the prevalence reported in Germany, Libya, and Greece (Table 7).

No significant difference was found between male and female children diagnosed with MIH, which is in agreement with other prevalence studies.^7^ However, female population showed a slightly high incidence of MIH as compared with male children, which is in agreement with the preceding studies.^7,9^ The prevalence of MIH is more severe in molars as compared with incisors. Mandibular molars and maxillary incisors are the commonly involved tooth, which is in concurrence with other preceding studies.^10^ In the present study, a strong correlation of MIH was observed with prenatal, perinatal, and postnatal infection/disorder in the children. A total of 125 of 191 children diagnosed with MIH had a history of postnatal infections, such as jaundice, chicken pox, cardiac disorder, kidney disorder, chest infection, vitamin A deficiency, high fever, long-term breastfeeding, and intake of antibiotics for long duration. Among all the aliments, chicken pox, vitamin A deficiency, and ear infections^11^ were the most common reasons for MIH in the present study. Varicella zoster virus and vitamin A deficiency cause abrupt changes or disturbances in epithelium-derived ameloblast, leading to hypomineralized enamel because of reduced secretory ability of ameloblast during enamel maturation.^12^ Similarly, antibiotic therapy during the first 3 years of birth may alter the secretory ability of ameloblast.^11^ Chest infection during the first year of life may create hypoxic conditions for ameloblast leading to MIH.^12^ A total of 9.9% of the diagnosed MIH children gave a history of high fever/fever of unknown origin. This is in agreement with a previous study^13^ as maternal pyrexia or high fever in the newborn can have disastrous effects on amelogensis ranging from enamel hypoplasia/hypomineralization to complete cellular degeneration. The study of 12 children diagnosed with MIH reported to have a history of long-term breastfeeding was in agreement with the findings of Alaluusua et al.^14^ This can be explained by the fact that some environmental hazardous elements (e.g., dioxins, mercury, pthalates) or the malnourished status of nursing mother may deprive the child of adequate nutrition leading to hypomineralization. On the contrary, Whatling and Fearne^15^ found no association regarding breastfeeding duration and MIH. Apart from a history of postnatal infections, 7 mothers reported to have gestational diabetes, 5 had hypertension, 12 had a history of intake of amoxicillin during pregnancy, 9 children had low birth weight (< 2.5 kg), while 7 accounted for preterm birth (< 7 months), constituting 40 children in total diagnosed with MIH. Molar incisor hypomineralization in 26 (13.6%) of the diagnosed children cannot be correlated with any of the known etiological factors or history of pregnancy/diseases in early childhood. Also type of delivery, i.e., cesarean and normal, did not have any significant impact on occurrence of MIH with the place of birth (urban or rural).

**Table 3:** **Table 3:** Distribution of affected molars and incisor with molar incisor hypomineralization by gender

		*Total (n* = *191)*				*Male (n = 92)*				*Female (n = 99)*					
		*No.*		*%*		*No.*		*%*		*No.*		*%*		*RR (95% CI), p-value*	
Molars		126		66.0		65		70.7		61		61.6		1.24 (0.88-1.73), 0.18	
Incisor with molar		53		27.7		16		17.4		37		37.4		0.54 (0.35-0.84), 0.002*	
Incisor with opacity		12		6.3		11		12.0		1		1.0		2.02 (1.60-2.56), 0.002*	

RR: Relative risk; CI: Confidence interval; *Significant

**Table 4:** **Table 4:** Distribution of number of first molars affected with molar incisor hypomineralization by gender

		*Total (n = 191)*				*Male (n = 92)*				*Female (n = 99)*					
		*No.*		*%*		*No.*		*%*		*No.*		*%*		*RR (95% CI), p-value*	
Single molar		72		37.7		34		37.0		38		38.4		0.96 (0.71-1.31), 0.83	
Two molars		53		27.7		23		25.0		30		30.3		0.86 (0.61-1.23), 0.41	
Three molars		40		20.9		16		17.4		24		24.2		0.79 (0.52-1.19), 0.24	
Four molars		14		7.3		7		7.6		7		7.1		1.04 (0.60-1.79), 0.88	

RR: Relative risk; CI: Confidence interval

**Table 5:** **Table 5:** Distribution of affected molars and incisors with type of delivery

		*Normal* *(n = 112)*				*Cesarean* *(n = 79)*					
		*No.*		*%*		*No.*		*%*		*RR (95% CI), p-value*	
Molars		75		67.0		51		64.6		1.04 (0.81-1.35), 0.72	
Incisor with molar		31		27.7		22		27.8		0.99 (0.76-1.30), 0.97	
Incisor with opacity		6		5.4		6		7.6		0.84 (0.47-1.50), 0.53	

RR: Relative risk; CI: Confidence interval

**Table 6:** **Table 6:** Distribution of number of affected first molars with type of delivery

		*Normal* *(n = 112)*				*Cesarean* *(n = 79)*					
		*No.*		*%*		*No.*		*%*		*RR (95% CI), p-value*	
Single molar		48		42.9		24		30.4		1.24 (0.98-1.56), 0.08	
Two molars		27		24.1		26		32.9		0.82 (0.61-1.11), 0.18	
Three molars		24		21.4		16		20.3		1.03 (0.77-1.37), 0.84	
Four molars		7		6.2		7		8.9		0.84 (0.49-1.44), 0.49	

**Table 7:** **Table 7:** Studies reporting prevalence of MIH around the world

*Place*		*Study*		*Sample* * size*		*MIH* *prevalence* *(%)*	
Sweden		Koch et al 1987		2,226		4-15	
Finland		Alaluusua et al 1996		102		17	
Switzerland		Clavadetscher 1997		1,671		6.4	
Turkey		Alpoz and Ertugrul		250		14.8	
		1999					
Sweden		Jalveik et al 2001		516		18	
Finland		Leppaniemi et al 2001		488		19	
The Netherlands		Weeheijm et al 2001		497		10	
UK		Zagdwon et al 2002		307		14.6	
Germany		Dietric et al 2003		2,408		6	
Denmark		Esmark et al 2003;		5,277		15-25	
		Weerheijim et al 2003					
Greece		Lygidakis et al 2004		2,640		6	
Slovenia		Kosem et al 2004		2,339		14	
Italy		Calderara et al 2005		227		13.7	
Germany		Preusser 2006		1,022		5.9	
Libya		Fteita et al 2006		378		2.9	
Lithuania		Jasulaityto et al 2007		1,277		9.7	
Bosnia		Muratbegovic et al		560		12.3	
		2007					
Kenya		Kemoli 2008		3,591		13.73	
Hong Kong		Cho et al 2008		2,635		2.8	
Bulgaria		Kukleva et al 2008		2,960		3.58	
Istanbul		Kusku et al 2008		147		14.9	
Wainuiomata		Mahoney et al 2009		522		14.9	
Jordon		Zawaideh et al 2011		3,666		17.6	
Argentina		Biondi et al 2011		1,098		15.9	
India		Present study		1,369		13.9	

MIH: Molar incisor hypomineralization

In the present study, 70.7% of males and 61.6% of females had hypomineralized molar, hence no significant difference was observed between affected molars and gender. However, involvement of both incisors and molars was significantly high in females (p = 0.002) as well as for females with incisor opacities (p = 0.002). It was observed that prevalence of affected single molar was high (37.7%), and 14 cases (7.3%) reported involvement of all four molars. Also the data in the present study revealed that higher the number of affected tooth, most likely is the severity of the lesions which can be explained by duration and time of insult during enamel formation. The finding of the present study is in accordance with the observation of Jalevik et al and Leppaniemi et al in 2001. Also the mean age of the affected children was 10.25 years in the present study, and it was observed that more severe lesion was present with increased age. The children in the age group of 8 to 9 years showed opacities or fewer breakdowns as compared with older children. This can be explained by the fact that a hypomineralized tooth after eruption is subjected to masticatory stress and caries attack leading to breakdown of tooth as the age increases, thus demanding need for more complex restorations. This finding supports the observation by Weerheijm et al that even minute opacities need years of follow-up to detect the breakdown as eventually it is uncertain which part of tooth will break and which will remain intact.

Comparing the MIH prevalence data among different countries, the present study showed a moderate prevalence. However, the actual comparison between the data is not possible due to difference in diagnostic criteria, sample size, and study design as some of the preceding studies laid stress on treatment needs, while others differentiated the severity of lesions. The limitation of the present study is paucity of literature and technologies to differentiate between opacities due to caries/trauma and MIH. This may lead to false-positive load of MIH in population when opacities are taken into consideration.

## CONCLUSION

The present study reports a prevalence of 13.9% in children of age group 8 to 12 years. Thus, MIH is a frequently occurring dental ailment in the pediatric population. It is an established fact from literature as well as the present study that any pre- and postchanges affecting ameloblast during enamel formation may lead to MIH. Hence, a protocol for early diagnosis and follow-up to access the squeal of breakdown should be undertaken along with parents and health workers. This may prevent the requirement of extensive restorative treatment, and preventive measures (pit fissure sealants, resin infiltrations) alone can reduce the severity of MIH. More studies in this field are needed to be undertaken to spread awareness in the general population and to practice specific preventive health care to the mother and child.
